# Michigan Dental Association

**Published:** 1877-07

**Authors:** E. S. Holmes

**Affiliations:** *Sec*. M. D. A.


### ARTICLE VIII.

### *Michigan Dental Association.*

The President, Dr. G. R. Thomas, has appointed the following Fellows of the Michigan Dental Association to fill the Standing Committees, viz:

*Anatomy and Histology.*—D. C. Hawxhurst, D. D. S., Battle Creek; E. G. Douglass and G. W. Stone, M. D.

*Physiology.*—Prof. J. A. Wattling, D. D. S., Ypsilanti; W. D. Tremper, D. D. S., and R. H. Tremper, D. D. S.

*Chemistry and Materia Medica.*—I. Douglass, D. D. S., Romeo; R. S. Bancroft and J. E. Post, D. D· S.

*Hygiene.*—B. T. Spellman, Detroit; J. Lathrop and H. H. Jackson.

*Pathology and Semeiology.*—J. A. Robinson, D. D. S., Jackson; G. H. Mosher, D. D. S., and Thomas Rix.

*Therapeutics.*—J. W. Finch, Adrian; M. H. Knapp, and W. P Morgan, D. M. D.

*Surgery.*—D. W. Smith, Jackson; W. H. Jackson, D. D. S., and E. Hunter.

*Education and Etiquette.*—E. S. Holmes, D. D. S., Grand Rapids; T. R. Perry and J. C Parker.

*Publishing.*—E. S. Holmes, D. D. S., Grand Rapids; G. R. Thomas, D. D. S., and W. D. Tremper, D. D. S.

*Executive.*—G. L. Field, D. D. S., Detroit; J. A. Watling, D. D. S., and D. W. Smith. With the President, Vice President, Secretary and Treasurer, ex-officio.

*Board of Censors.*—Prof J. A. Watling, D. D. S., for one year; D. C. Hawxhurst, D. D. S., for two years; W. H. Jackson, D. D. S., for three years.

*Consulting and Visiting.*—G. R. Thomas, D. D. S., for one year; A. T. Metcalf, D. D. S., for two years; B. T. Spellman, for three years.

These gentlemen are expected to be prepared, and to report, either in person or through the Chairman of their respective committees on some point of the general subject assigned them, at the next session of the Michigan Dental Association, to be held at Ann Arbor on the 9th day of October, 1877, at 2 o'clock P. M.

The Secretary would respectfully suggest the following points for discussion by the several committees, viz: *Anatomy, &c.* The Anatomy and Histology of the dental pulp

*Physiology.*—The provisions of nature for the protection of the dental pulp threatened with disease, and to what extent can they be relied on.

*Chemistry and Materia Medica.*—The chemical constituents, and medical properties of Lacto Phosphate of Lime.

*Hygiene.*—The prophylactic measures best adapted to prevent the consumption of the dental organs.

*Pathology and Semeiology.*—The symptoms of Exodontosis (dental exostosis), and the nature of the affection.

*Therapeutics.*—The medicinal remedies for Dental Neuralgia.

*Surgery.*—The surgical treatment of the Dental Pulp, diseased by the decomposition of the dentinal tissues.

There are other very important subjects to be considered at this meeting, and it is hoped and expected that every Member of the Association will be present, and prepared to do his duty.

A general invitation is hereby extended to all dentists—and every one interested in dental progress—to be present.

E. S. Holmes, *Sec.* M. D. A.

---

